# CAR-T细胞治疗后噬血细胞综合征2例报告并文献复习

**DOI:** 10.3760/cma.j.issn.0253-2727.2023.05.012

**Published:** 2023-05

**Authors:** 敏 喻, 倩 张, 繁聪 孔, 玉兰 周, 菲 李

**Affiliations:** 南昌大学第一附属医院血液科，江西省血液病临床医学研究中心，南昌大学淋巴肿瘤疾病研究所，南昌 330006 Department of Hematology, The First Affiliated Hospital of Nanchang University, Institute of Hematology, Academy of Clinical Medicine of Jiangxi Province, Institute of Lymphoma of Nanchang University, Nanchang 330006, China

嵌合抗原受体T细胞（CAR-T）疗法在多种血液系统恶性肿瘤中展现了显著的治疗效果，多项商品化CAR-T产品获批上市，开拓了肿瘤免疫治疗的新时代[1]–[3]。尽管CAR-T疗法疗效显著，其在临床治疗过程中经常伴随不同的不良反应，噬血细胞性淋巴组织细胞增生症（HLH）/巨噬细胞激活综合征（MAS）是一组病理性免疫激活引起过度炎症反应为特征的临床综合征，病情凶险，死亡率高[4]。据报道，接受CAR-T治疗的小部分患者治疗过程中会出现HLH，不及时治疗往往导致快速死亡[5]。本文主要报道2例CAR-T治疗引起HLH的病例并进行文献复习，以期增强临床医师对CAR-T相关HLH的认识，为临床早期识别和及时治疗提供参考。

## 病例资料

例1，女性，45岁。2021年1月诊断费城染色体阳性急性淋巴细胞白血病（Ph^+^ ALL），根据《中国成人急性淋巴细胞白血病诊断与治疗指南（2016年版）》[6]，使用VICLP（柔红霉素、长春新碱、环磷酰胺、地塞米松、培门冬酶）联合伊马替尼靶向治疗获得完全缓解（CR），其后进行CAM（环磷酰胺、阿糖胞苷、硫唑嘌呤）、HD-MTX（大剂量甲氨蝶呤）+L-Asp（培门冬酶）和IA（伊达比星、阿糖胞苷）方案联合伊马替尼强化巩固治疗，疗效评估均为CR。2021年7月复查骨髓细胞学示ALL复发，检出的激酶区突变p.F359I，可能跟疾病早期复发进展密切相关。2021年8月患者在本院参加人源化抗CD19 CAR-T细胞（二代，共刺激分子为4-1BB）输注的临床研究，单采外周血T细胞后予尼洛替尼联合VCP（环磷酰胺、地塞米松、长春新碱）方案桥接治疗，FC方案（氟达拉滨+环磷酰胺）清淋化疗前复查骨髓细胞形态学示原始淋巴细胞占35％，化疗完成后2 d回输CAR-T细胞1.2×10^6^/kg。回输后6 h患者出现发热（38.6 °C）、肌肉酸痛等非特异性症状，给予补液、对乙酰氨基酚口服退热，次日患者仍持续高热（39.5 °C），缺氧（未吸氧状态学血氧饱和度90％），低血压（90/50 mmHg，1 mmHg＝0.133 kPa）和双下肢水肿，CRP（108 mg/L）、铁蛋白（SF，2 208 µg/L）和细胞因子（IFN-γ、TNF-ɑ、IL-6）显著升高，考虑Ⅲ级细胞因子释放综合征（CRS），先后予3次托珠单抗（8 mg/kg）和地塞米松（10 mg/d×3 d）处理，患者体温逐渐下降至正常，缺氧和低血压等症状改善。CAR-T回输后第10天，患者再次出现持续性高热（39.5 °C）伴肝功能损伤（ALT 450 U/L、AST 560 U/L），高甘油三脂血症（5.6 mg/L），SF上升至11 078 µg/L，IL-6上升至936.1 ng/L，sCD25上升至10 650 U/ml，纤维蛋白原下降至0.8 g/L，WBC与PLT显著降低，骨髓可见噬血现象，诊断继发性HLH，在继续使用托珠单抗拮抗IL-6基础上增加地塞米松剂量（10 mg每12 h 1次），48 h后患者仍有发热，SF最高上升至227 070 µg/L，继续增加地塞米松剂量（10 mg每6 h 1次，3 d），联合芦可替尼（10 mg每12 h 1次，7 d），同时予抗感染、成分输血和护肝等治疗，1周余后患者HLH指标显著好转，WBC进行性升高，外周血出现幼稚细胞，复查骨髓判断疾病未缓解，最终因疾病进展死亡。

例2，男性，35岁。2018年2月因腹腔包块就诊，诊断“B细胞非霍奇金淋巴瘤”，先后行利妥昔单抗联合HyperCVAD（环磷酰胺、长春新碱、多柔比星、地塞米松、甲氨蝶呤、阿糖胞苷）、CODOX（环磷酰胺、长春新碱、多柔比星、阿糖胞苷）等方案化疗，期间行脊髓鞘内注药预防中枢神经系统浸润，总体治疗过程顺利。患者结束4个疗程化疗后不久出现多处皮肤散在包块，经穿刺活检确诊疾病复发进展，2018年8月患者在本院参加抗CD19 CAR-T细胞（二代，共刺激分子为4-1BB）输注的临床研究。2018年9月19日回输CD19 CAR-T细胞2×10^6^/kg，回输后第1～6天持续发热，体温进行性升高伴全身水肿，窦性心动过速（每分140次），呼吸急促（每分35次），对乙酰氨基酚无法使体温降至正常，细胞因子显著升高，合并心功能不全、消化道出血、代谢性酸中毒、凝血功能异常等严重并发症，考虑为Ⅳ级CRS，予6次托珠单抗（8 mg/kg）、地塞米松（10 mg每12 h 1次，4 d）和血浆置换清除炎症因子等治疗，症状较前缓解，皮肤包块明显消退，彩超提示腹部包块较前显著缩小，判断治疗有效。CAR-T回输后2周，患者再次出现持续性高热伴嗜睡、反应迟钝，癫痫发作1次（给予抗癫痫和降颅压处理后症状稍缓解），复查细胞因子水平极度升高，IL-6达1 200 ng/L，sCD25达120 000 U/ml，考虑CRS合并神经毒性，在继续使用托珠单抗（8 mg/kg）基础上联合地塞米松（15 mg每12 h 1次），患者症状仍进行性恶化，细胞因子水平极度升高，SF高达18 000 µg/L，结合血象明显低下、肝肾功能损害、低纤维蛋白原、高甘油三脂血症等表现，符合继发性HLH诊断标准，在接受HLH治疗前因神经症状进行性恶化死亡。

## 讨论及文献复习

CRS是CAR-T最常见的严重不良反应之一，中重度CRS临床表现和实验室检查与HLH极为相似，使得在CRS背景下HLH的诊断难度增加，截然划分二者十分困难。Kim等[7]使用HLH-2004和H-score对复发难治大B细胞淋巴瘤CAR-T治疗后继发HLH的患者进行评价，发现传统上述标准特异性存在明显不足，无法将HLH从CRS中区分。有学者认为大部分中度CRS患者即能符合经典HLH-2004标准，HLH指标往往随CRS缓解而恢复，因此没有必要专门制定CAR-T治疗相关HLH诊断标准。MD Anderson癌症中心Neelapu等[8]认为CAR-T相关HLH是较CRS更高级别系统性炎症反应，治疗需要更积极的免疫抑制，重新定义诊断标准有助于筛选这部分患者进行迅速干预，降低死亡率。他们制定了CAR-T治疗相关HLH诊断的新标准，CRS期间出现SF>10 000 µg/L，同时合并≥3级的器官毒性（肝、肾、肺），骨髓或其他器官中可见噬血现象，则应诊断为CAR-T相关HLH，若合并2个及以上器官毒性，即使骨髓或组织未见噬血现象，诊断仍应成立。根据上述标准，Neelapu等[9]报道接受CAR-T治疗患者中HLH发生率低至1％。然而，在一项美国国家癌症研究所Shah教授领衔的抗CD22 CAR-T细胞疗法的Ⅰ期临床研究中观察到，19例（38％）患者可以诊断CAR-T治疗相关HLH（Neelapu标准），揭示了CD22 CAR-T细胞一种独特的毒性特征[10]。这一现象在CD4/CD8 T细胞选择组更明显，提示T细胞载体的选择和制备工艺等因素十分重要，可以显著影响产品毒副作用。

根据Neelapu标准，本组2例患者均符合CAR-T治疗相关HLH诊断：其中例1从第10天开始SF>10 000 µg/L，合并Ⅲ级肝脏毒性，并在第12天进行了骨髓抽吸物中噬血细胞的组织学确认；例2两周后SF>10 000 µg/L，伴显著肝肾功能损害。例1 CAR-T治疗期间出现明显HLH表现，复查骨髓示大量原始淋巴细胞，不排外肿瘤进展引起HLH；例2治疗期间皮肤浸润肿块出现明显消退，外周血检测出显著CAR-T扩增高峰，因此该患者HLH应该归因于CAR-T细胞毒性而非恶性肿瘤。如果出现意识障碍、头晕、头痛、癫痫发作等异常症状时，需要考虑HLH中枢神经系统受累可能。例2嗜睡和癫痫表现很难区分CAR-T神经毒性或HLH累及神经系统，积极完善脑脊液检查和头颅影像学等检查可能有助于区分。值得注意的是，Neelapu等[8]认为CAR-T相关HLH大多发生在CAR-T输注5 d内，常继发于严重未受控制的CRS。然而本组2例患者HLH均发生于输注后近2周，并且跟CRS高峰无明显重叠表现。迟发性HLH并不罕见，Lichtenstein等[11]回顾性分析了CD22 CAR-T治疗复发难治ALL临床试验数据，发现HLH事件仅在CRS患者中发生且发病延迟，输注前NK细胞亚群计数下降和骨髓T/NK细胞比值升高是CAR-T相关HLH发生的高危因素。NK细胞减少/功能缺陷，导致CAR-T增殖抑制因素减少，CAR-T扩增持续进一步削弱NK细胞计数和功能，二者互相作用形成恶性循环，巨噬细胞显著活化，诱发了HLH，这跟既往认识的HLH发病机制较为一致。穿孔素/颗粒酶是CTL细胞杀伤靶细胞的重要途径，其基因突变或蛋白表达缺陷可引起原发HLH，来自小鼠的研究证实，当CAR-T出现穿孔素缺陷时，小鼠治疗模型重现了经典HLH现象，提示CAR-T细胞功能缺陷会引起免疫应答失控，引起机体持续炎症反应，导致HLH发生[12]。上述机制均可一定程度解释CAR-T治疗过程中迟发性HLH的发生。

CAR-T治疗相关HLH目前尚无标准治疗方案，临床上多采用托珠单抗和糖皮质激素。关于糖皮质激素的剂量，应当参照高级别CRS的处理，地塞米松10 mg每6 h 1次或甲泼尼龙1 g/d，必要时继续加大剂量，糖皮质激素使用至症状缓解后快速减量，尽早使用糖皮质激素类药物可以使得高级别炎症反应发生可逆性缓解[8]。有研究发现，糖皮质激素的剂量、持续时间和使用时机显著影响CAR-T细胞治疗的长期疗效[13]。然而，ZUMA-1最新队列数据提示，早期/预防性应用糖皮质激素和托珠单抗可以更好管理CRS和神经毒性，并不影响CAR-T在体内的扩增和疗效[14]–[15]。如何合理运用糖皮质激素还有待进一步临床研究，最终的目标是保持CAR-T最大疗效的同时防止发生危及生命的炎症反应。Neelapu等[8]建议，除了使用托珠单抗、糖皮质激素及对症治疗，HLH症状48 h内未缓解应使用依托泊苷（75～100 mg/m^2^，4～7 d后可重复使用），其可选择性清除激活的T细胞并抑制炎性细胞因子产生。但是，目前罕有研究在CAR-T相关HLH中使用依托泊苷，因其不可避免清除机体CAR-T细胞，影响潜在治疗效果。尽管如此，如果HLH爆发起病合并多器官功能损害，依托泊苷的应用仍非常必要。HLH和CRS都属于类似的系统性高炎症反应，但HLH炎症因子谱常广于CRS，后者以IL-6升高为主，常呈暂时性升高，相关症状可通过托珠单抗改善，HLH通过上述手段很难取得理想效果。IL-1在系统炎症反应的病理生理学中具有重要作用，在治疗方案中加入抑制IL-1的阿纳金拉（Anakina）可能有效控制CAR-T相关HLH[16]–[18]。HLH的高细胞因子血症可降低CD8^+^ T细胞的凋亡潜能，导致地塞米松耐药，芦可替尼可恢复CD8^+^T细胞的凋亡潜能，逆转地塞米松耐药[19]。有研究评估芦可替尼用于CAR-T治疗中糖皮质激素耐药的CRS，患者症状明显缓解，CAR-T细胞功能未受到影响[20]。本组例1通过芦可替尼控制HLH，也取得了不错的效果，提示芦可替尼联合地塞米松用于控制CAR-T相关HLH极具应用前景。此外，IFN-γ抑制剂（伊帕伐单抗）、CD52单抗（阿伦单抗）等新药也可能有一定的疗效[8],[21]。药物之间联合使用可能具有协同作用，其理想的药物组合、给药顺序及剂量，以及整体免疫抑制的强度等均值得进一步研究探索。
